# Hyperstoichiometric Interaction Between Silver and Mercury at the Nanoscale**

**DOI:** 10.1002/anie.201106776

**Published:** 2012-02-03

**Authors:** Kseniia V Katok, Raymond L D Whitby, Takahiro Fukuda, Toru Maekawa, Igor Bezverkhyy, Sergey V Mikhalovsky, Andrew B Cundy

**Affiliations:** Nanoscience & Nanotechnology Group, Faculty of Science and Engineering, University of BrightonLewes Road, Brighton, BN2 4GJ (UK); Nazarbayev University53, Kabanbay Batyr Avenue, Astana (Kazakhstan); Bio-Nano Electronics Research Centre, Toyo University2100 Kujirai, Kawagoe Saitama 350-8585 (Japan); Laboratoire Interdisciplinaire Carnot de Bourgogne UMR5209 CNRS-Universite de Bourgogne, 9 av A. Savary, BP 47870, 21078 Dijon Cedex (France)

**Keywords:** hyperstoichiometry, mercury, nanoparticles, redox chemistry, silver

Extraction of heavy metals (e.g. silver, gold) through the formation of amalgams with mercury has been utilized in jewellery production^1^ and mining^2^ for over 2500 years. This has been contemporarily applied in a nanoformulation for the opposite process with the abstraction of mercury from waste sources using zero-valent nanoparticles of noble metals.^3^ The interaction of metal nanoparticles with other species has shown great promise for a number of applications^4^ and in particular silver nanoparticles (AgNPs) have been used as an anti-microbial agent in textiles and composites^5^ and also (less frequently) for the destruction of pesticides or the removal of mercury from industrial effluents and other waters.^6^ With the formation of silver through a silicon hydride reduction, we expect that as the size of the particle is reduced to the nanoscale its interaction with aqueous mercury will be dominated by surface forces.

It is well known that at the bulk scale, aqueous mercury(II) interacts with silver metal(0) with a stoichiometric ratio of 1:2 [Eq. (1)], resulting in zero-valent mercury.


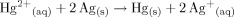
(1)

Herein we report that as the diameter of AgNPs is reduced below 32 nm, mercury(II) is reduced from water onto AgNPs with the mercury-to-silver ratio reaching 1.125:1 for 11 nm AgNPs. Moreover, silver is not oxidized into solution, rather, Ag–Hg solid amalgams are rapidly formed, immobilized on an inert silica substrate. This effect promises new insights to nanoscale chemistry and significant advancements in applications for wastewater purification, enhanced chemical catalysis, and toxicity of nanoscale systems.

Current approaches to the generation of AgNPs use chemical reducing agents and stabilizers, which result in residual groups on the surface of AgNPs, for example, carboxylic or citrates.^7^ Such groups promote electrostatic–ionic attractions between the nanoparticle surface and heavy metal ions.^6^ Metal particles have high surface energy and the reduction of AgNP size incurs a further increase in their surface energy causing their agglomeration and thus limiting the available surface area for sorption. To control their size, we generated AgNPs discretely separated on the surface of a modified silica substrate containing silicon hydride groups ≡SiH. Silicon hydride groups anchored to the surface of silica particles possess weak reducing properties,^8^ which are sufficient for generating “chemically pure” zero-valent silver^9^ by the reduction of silver cations according to Equation (2) (see Supporting Information, Figure S2).^10^


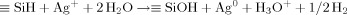
(2)

By adjusting the surface density of silicon hydride groups and the incubation time with a solution of silver nitrate, it was possible to control the size of the AgNPs formed on the silica surface (Figure 1 a–c). Although the primary interaction between the silicon hydride groups and silver cations yields zero-valent Ag atoms, the final metal deposition occurs in a form of much larger nanoparticles, thus we postulate that the mechanism of AgNP formation incorporates nucleation, growth, Ostwald ripening (also called coarsening), and aggregation processes.^11^

**Figure 1:**
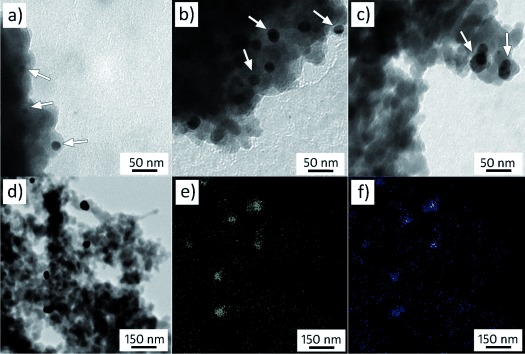
Modification of C-120 type silica (specific surface area 114 m^2^ g^−1^) using triethoxysilane generates silicon hydride groups. These are subsequently used to reduce a silver nitrate solution to AgNPs, which are affixed to the top of silica. TEM images reveal the distribution of near-spherical AgNPs, appearing as darker contrast particles (white arrows) against the fused silica substrate, with particle sizes averaging a) 11 nm, b) 31 nm, and c) 45 nm. d) EDX mapping analysis reveals the corresponding location of silver and mercury in the TEM image for the e) Ag (L_α_ peak) and f) Hg (M_α_ peak) after their reaction and shows that the distribution of Hg correlates only to the location of AgNPs.

Their separation from neighboring AgNPs on the silica substrate ensures that a greater proportion of the surface of the silver is available for interaction with its local environment, as compared with an agglomeration of AgNPs. Extensive washing of silver-modified silica resulted in no leaching of the AgNPs, confirming that they are stably immobilized on the substrate. Moreover, according to FTIR analysis all silicon hydride groups have reacted and oxidized to silanol groups (Figure S2).

The silicon hydride groups have a significant advantage over use of chemical agents in that the final silver is free of any residual groups and lends itself to the direct formation of amalgams with mercury. Once AgNPs were generated on silica, Hg^II^ was successfully removed from solution. TEM-EDX analysis (transmission electron microscopy-energy dispersive X-ray) revealed that mercury is only located on the silica at the sites of AgNPs (Figure 1 d–f and S2) confirming the Ag–Hg interaction. In the absence of AgNPs, no mercury was detected on silica across the pH range tested, demonstrating that silanol or pores within silica do not account for the vast volume of mercury removed.

Mercury adsorption isotherms were obtained for all materials (Figure 2 a) and the maximum adsorption mercury loading (*A*) was calculated from the linearized Langmuir equation [Eq. (3)],


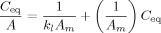
(3)

**Figure 2:**
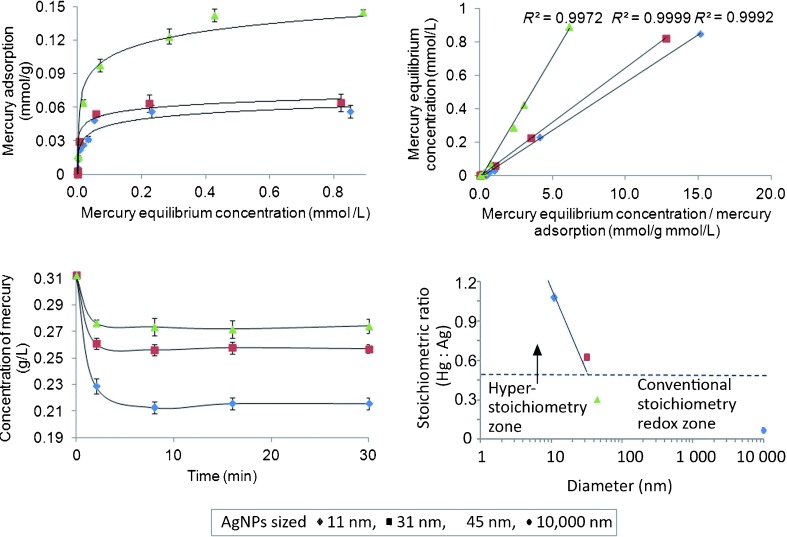
a) Hg^II^ adsorption isotherms on silica with AgNPs were performed in batch with 0.1 g of the three silver-containing silica samples per 40 mL of Hg(NO_3_)_2_ solution with concentrations ranging from 0.15 to 312 mg L^−1^ in the pH range of 4 to 7. The time allowed for establishing equilibrium was 120 min with constant shaking. The solution was separated by filtration and the residual mercury concentration was determined using inductively coupled plasma mass spectrometry (ICP-MS). b) Adsorption isotherms for the maximum adsorption of mercury onto different sized and quantities of AgNPs on silica were fitted to a Langmuir adsorption equation with *R*^2^ values above 0.99. c) The kinetics of Hg^II^ uptake, starting with a 312 mg L^−1^ stock solution, was studied for three different AgNP sizes on silica over 30 min. d) Ag rods, 0.01 mm in diameter and 5 mm in length, exhibited removal of Hg^II^ with a stoichiometric ratio of Hg^II^ to Ag^0^ below 0.5, but when using AgNPs this ratio increases from 0.29:1 up to the maximum of 1.125:1 when the Ag particle size is reduced from 45 nm to 11 nm. The change from conventional to hyper-stoichiometry occurs around 32 nm.

where *A_m_* and *k_l_* are the characteristic Langmuir parameters related to the maximum adsorption capacity and the intensity of adsorption, respectively. The adsorption profile fits the Langmuir equation with a high degree of accuracy (Figure 2 b), indicating that the adsorption mechanism does not change for AgNPs when varying their size from 11 to 45 nm. When analyzing the ratio of mercury removed from solution to the amount of silver present, it was found that the smaller sized AgNPs on silica possessed a higher capacity for mercury binding and a higher rate of mercury removal from solution (Figure 2 c), which corresponds to a previously unreported and unusually high mercury-to-silver stoichiometric ratio (Table 1).

**Table 1:** Summary of analysis of mercury adsorption on different sized silver particles.

System	Average diameter of AgNPs [nm]	Ag content (mmol of Ag/ g SiO_2_)	Maximum Hg loading (mmol of Hg/ g SiO_2_)	Stoichiometric ratio of Hg^II^ to Ag^0^
C120-Ag-5	11	0.050	0.056	1.125:1
C120-Ag-10	31	0.100	0.064	0.64:1
C120-Ag-40	45	0.500	0.145	0.29:1
Ag rod	10 000^[a]^	–	–	0.07:1^[b]^

[a] The size of the Ag rod was 0.01 by 5 mm. [b] This ratio was taken from the quantity of Hg^II^ reduced from solution with that of Ag^I^ oxidized into solution.

We confirmed the bulk-scale effect using a silver rod. Examining the solution after the reaction using ICP-MS, we found 4.3×10^−3^ mmol of Ag^I^ in solution compared with 2.9×10^−4^ mmol of Hg^II^ removed from solution, which demonstrates that the system behaves according to the conventional understanding of a redox reaction between Hg^II^ and Ag^0^ [Eq. (1)] with a ratio of 0.07:1.0 (Figure 2 d under the horizontal broken line). However, when the Ag particle size is reduced below a critical size, ca. 32 nm (according to linear extrapolation in the hyperstoichiometry zone, Figure 2 d), the quantity of Hg^II^ removed from solution is far greater than the amount of Ag^I^ released into solution.

In the system containing silica with 11 nm AgNPs, the solution at equilibrium contained 7×10^−4^ mmol of Ag^I^ ions released from the silica substrate after reaction with mercury(II), compared with the adsorption of 0.056 mmol of Hg^II^ from solution. This effect, which we call hyperstoichiometry, is solely dependent on the size of AgNPs, therefore the ratio of Hg^II^ coming out of solution is compared with the initial quantity of Ag^0^ available that drives the reaction rather than compared with the resulting Ag^I^ in solution, otherwise the stoichiometry ratios would be far higher. We have eliminated various parameters that might affect stoichiometry, namely pH, residual silicon hydride and silanol groups, light reduction of hydrated silver, and contaminants within the materials and solutions. The hyperstoichiometry effect has been confirmed for both mercury nitrate and mercury acetate, the latter exhibited a 1.7:1 ratio (Hg to Ag) for ca. 10 nm AgNPs, and will be evaluated for other anion species. It has been found that Ag^+^ can adsorb to AgNPs^12^ and would therefore be undetectable by ICP-MS. Therein, Ag^+^ could be reduced by the anion (see Supporting Information) to Ag^0^ and partake in further reduction of Hg^2+^ to Hg^0^. It is apparent that the hyperstoichiometric ratio is linked to the size of the AgNPs and it is therefore surmised that the smaller AgNPs have superior release and catalytic recycling of the Ag^+^ released into solution, possibly driven by the greater surface energy of nanosized silver particles over its bulk scale counterparts.

The powder X-ray diffraction (XRD) profile of AgNP on silica is consistent with that of silver metal possessing a face-centred cubic lattice (Figure 3 a, line 1). During its interaction with mercury, no crystal phase transformation of silver was observed (Figure 3 a, lines 2 and 3), but a progressive decrease of the diffraction intensity of the silver peaks was found with an increasing amount of mercury adsorbed by the system. Additional diffraction peaks emerged (Figure 3 a, lines 3 and 4 asterisks), which correspond to the formation of the Schachnerite amalgam (Ag_1.1_Hg_0.9_), thus demonstrating that Hg^II^ has reduced to Hg^0^ and Ag^0^ is still present to facilitate a direct interaction between the metals. At higher loadings of mercury, no crystal phases were detected (Figure 3, line 5) indicating the formation of a non-crystalline state. This can be clearly seen under TEM observations where the average particle size of AgNPs has increased on contact with mercury (Figure 3 c, e and S3–4), which accompanies the disappearance of the crystal lattice fringes of silver (Figure 3 d, f).

**Figure 3:**
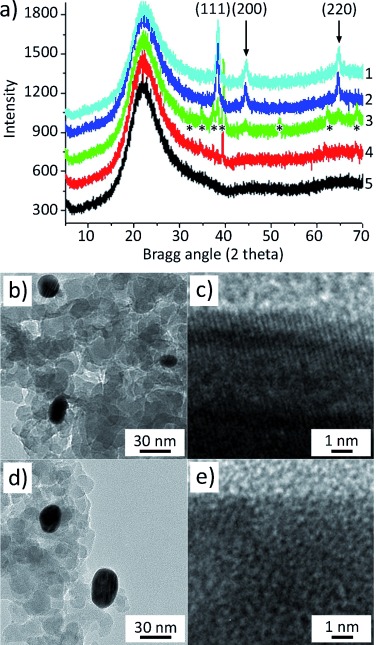
Silica samples containing 11 nm AgNPs were shaken with 40 mL of Hg(NO_3_)_2_ solution of increasing concentration for 120 min at pH 7. The solution was separated by filtration and the solid washed with deionized water then dried. a) Powder XRD patterns were recorded for the final Ag–Hg amalgams on silica for concentrations 0.15 (line 1), 1.56 (line 2), 15.6 (line 3), 39 (line 4) to 78 mg L^−1^ (line 5). b) TEM reveals 11 nm AgNPs on silica before interaction with mercury and c) the crystal lattice structure revealed under high magnification. d) After interaction with mercury, TEM images show an increase in size of AgNPs due to the formation of a Ag–Hg amalgam, which e) exhibits no crystallinity.

The final composite was analyzed by using X-ray photoelectron spectroscopy (XPS) (Figure S5) and high-resolution TEM, but the results revealed that the levels of mercury were lower than calculated from the ICP-MS measurements, which we assign to its evaporation under the application of high vacuum required by these characterization methods. EDX analysis within TEM (Figure 1 e,f) indicated an elemental composition ratio of Hg to Ag close to 1:1. Therefore, we dissolved the metal fractions from the final composite sample of 11 nm AgNPs–Hg amalgam in concentrated nitric acid and the liquid was then analyzed by ICP-MS, which revealed a ratio of Hg to Ag of 0.7:1. Herein, the slightly lower than expected ratio arises as the level of dissolved silver approached the lower detection limit of the ICP-MS, but demonstrates that without the application of a vacuum, the level of Hg^II^ adsorbed into AgNPs through the hyperstoichiometry effect is high.

At present it is difficult to suggest the exact structure of the Ag–Hg composite in the AgNPs. Taking into account that the atomic radii of Ag (160 pm) and Hg (150 pm) are similar, it is possible to estimate the “kissing”, or the Newton number, of spheres that can simultaneously touch the central sphere of the same radius. For a 2-D system the number is 6 and for a 3-D system the number is 12.^13^ As AgNPs would have both 2-D (adjacent to the surface) and 3-D dimensions, the maximum number of Hg atoms around each Ag atom could be between 6 and 12. In bulk systems such a high ratio between Hg and Ag can be only achieved for liquid amalgams. The fact that the Ag–Hg composites obtained using AgNPs are solid suggests that certain properties of the system have changed significantly at the nanoscale dimensions. The diffusion coefficients (*D*) of Hg in its amalgams vary widely from 10^−10^ cm^2^ s^−1 [14]^ to 10^−13^ cm^2^ s^−1^.^15^ Assuming that *D* is 10^−10^ cm^2^ s^−1^, it is possible to estimate the diffusion path of a mercury atom within a spherical solid Ag particle to be ca. 5 nm within 3 min, which is the time chosen according to the experimental data on adsorption kinetics presented in Figure 2 c. This result qualitatively explains the fastest adsorption kinetics for the smallest AgNPs and the reduction of the hyperstoichiometric ratio with the increase of NP size.

The AgNPs generated herein are deemed to be free of residual chemical groups, which enable surface effects to predominate in their physicochemical properties. Further experiments will be carried out to confirm the anion reduction effect by use of mercury chloride, which would prevent the recycling of Ag^+^ through the precipitation of AgCl. Ultimately, we show that at the nanoscale a hyperstoichiometry effect between the aqueous mercury and AgNPs occurs.

## Experimental Section

Silica matrices were modified with triethoxysilane in the presence of acetic acid under reflux (2 h, 90 °C) in order to graft silicon hydride groups (≡SiH) onto the silica surface (C-120-H). Dried modified silica was then stirred with AgNO_3_ at room temperature, which generate AgNPs on the silica surface. Samples were then dried for 24 h at 150 °C. All batch sorption experiments were performed with silica containing either 0.05, 0.10, or 0.44 mmol of silver, where typically 0.1 g of silver-containing silica was placed in a conical flask and 40 mL of a pre-determined concentration of Hg(NO_3_)_2_ was added. The mixture was shaken for 120 min at room temperature to allow mercury to adsorb on the Ag-loaded silica. After soaking, the resultant solid was filtered and dried in an air environment. Solutions were analysed using ICP-MS.

## Supporting Information

Detailed facts of importance to specialist readers are published as ”Supporting Information”. Such documents are peer-reviewed, but not copy-edited or typeset. They are made available as submitted by the authors.

## References

[1] Hesse RW (2007). Jewelrymaking through History.

[2] Lacerda L, Salomons W (1998). Mercury from Gold and Silver Mining: A Chemical Time Bomb?.

[3] Bootharaju MS, Pradeep T (2010). J. Phys. Chem. C.

[4a] Yavuz CT, Mayo JT, Yu WW, Prakash A, Falkner JC, Yean S, Cong LL, Shipley HJ, Kan A, Tomson M, Natelson D, Colvin VL (2006). Science.

[4b] Wasan DT, Nikolov AD (2003). Nature.

[4c] Shannon MA, Bohn PW, Elimelech M, Georgiadis JG, Marinas BJ, Mayes AM (2008). Nature.

[4d] Yuan JK, Liu XG, Akbulut O, Hu JQ, Suib SL, Kong J, Stellacci F (2008). Nat. Nanotechnol.

[5a] Alt V, Bechert T, Steinrucke P, Wagener M, Seidel P, Dingeldein E, Domann E, Schnettler R (2004). Biomaterials.

[5b] Lee HJ, Jeong SH (2005). Text. Res. J.

[5c] Xu XY, Yang QB, Wang YZ, Yu HJ, Chen XS, Jing XB (2006). Eur. Polym. J.

[6] Pradeep T, Anshup (2009). Thin Solid Films.

[7] Luo GQ, Yao H, Xu MH, Cui XW, Chen WX, Gupta R, Xu ZH (2010). Energy Fuels.

[8] Müller R (1950). “Über Silicone (I). Zur angewandten Chemie der Silicone (Synthese)”. Chem. Tech.

[9] Tertykh VA, Katok KV, Yanishpolskii VV (2008). Russ. J. Phys. Chem. A.

[10] Reed-Mundell JJ, Nadkarni DV, Kunz JM, Fry CW, Fry JL (1995). Chem. Mater.

[11] Harada M, Katagiri E (2010). Langmuir.

[12] Liu J, Hurt RH (2010). Environ. Sci. Technol.

[13] Conway JH, Sloane NJA (1993). Sphere Packings, Lattices, and Groups.

[14] Lee KH, Shin MC, Lee JY (1986). J. Mater. Sci.

[15] Malhotra ML, Reynolds L, Barker RE (1974). J. Chem. Phys.

