# Supplementary material for: Hyperstoichiometric Interaction Between Silver and Mercury at the Nanoscale
Source: Angew Chem Int Ed Engl. 2012 Feb 3;51(11):2632–5. doi: 10.1002/anie.201106776 (PMC3380547; doi:10.1002/anie.201106776)
Supplement: Supplementary file 1 [file anie0051-2632-SD1.pdf]

Angewandte Chemie
Eine Zeitschrift der Gesellschaft Deutscher Chemiker

Supporting Information
© Wiley-VCH 2012
69451 Weinheim, Germany

# Hyperstoichiometric Interaction Between Silver and Mercury at the Nanoscale**

*Kseniia V. Katok, Raymond L. D. Whitby,* Takahiro Fukuda, Toru Maekawa, Igor Bezverkhyy, Sergey V. Mikhalovsky, and Andrew B. Cundy*

anie_201106776_sm_miscellaneous_information.pdf

**– Abbreviations –**

| | |
|---|---|
| AgNP | Silver Nano Particle |
| EDX | Energy Dispersive X-ray |
| FTIR | Fourier Transform Infra Red |
| ICP-MS | Inductively Coupled Plasma Mass Spectroscopy |
| TEM | Transmission Electron Microscopy |
| XRD | X-Ray Diffraction |

## S1. Introduction

Silica can be modified to incorporate silicon hydride groups, which can reduce silver ions to their zero valent metal form. By controlling the reaction parameters, it was possible to control the size of the silver down to the nanoscale. Silver can form redox reactions with mercury with a stoichiometry ratio of 1 : 2 (Hg : Ag), however, as the size of the particle is reduced below 32 nm, this ratio increases and solid state Ag-Hg amalgams result (Fig. S1). It was found that the ratio of mercury removed from solution to the amount of silver present prior to reaction dramatically increases with a decreasing size of silver particles, thus achieving hyperstoichiometry.

## S2. Transformation of silica

In the formation of AgNPs, silicon hydride groups are first generated on the surface of silica according to the methods summary. After modification, the stretching vibration of O–H bonds of the surface silanol groups at 3750 $cm^{-1}$ (Fig. S2a, curve 1) was not observed in the IR spectrum. Instead an intense band with an absorption maximum at 2240 $cm^{-1}$ was observed (Fig. S1a, curve 2), which corresponds to the stretching vibration of the ≡Si–H bond, denoting that a number of silanol groups were substituted with silicon hydride groups. After reaction with silver nitrate, the band corresponding to the ≡Si–H groups denoting complete reaction of silicon hydride groups (Fig. S2c). Their presence was also confirmed using iodometric back-titration of excess of iodine with sodium thiosulfate, which indicated that 5, 10 and 44 mmol of silicon hydride groups per gram of silica were generated, denoted as C-120-Hyride-5, C-120-Hydride-10 and C-120-Hydride-44 respectively.

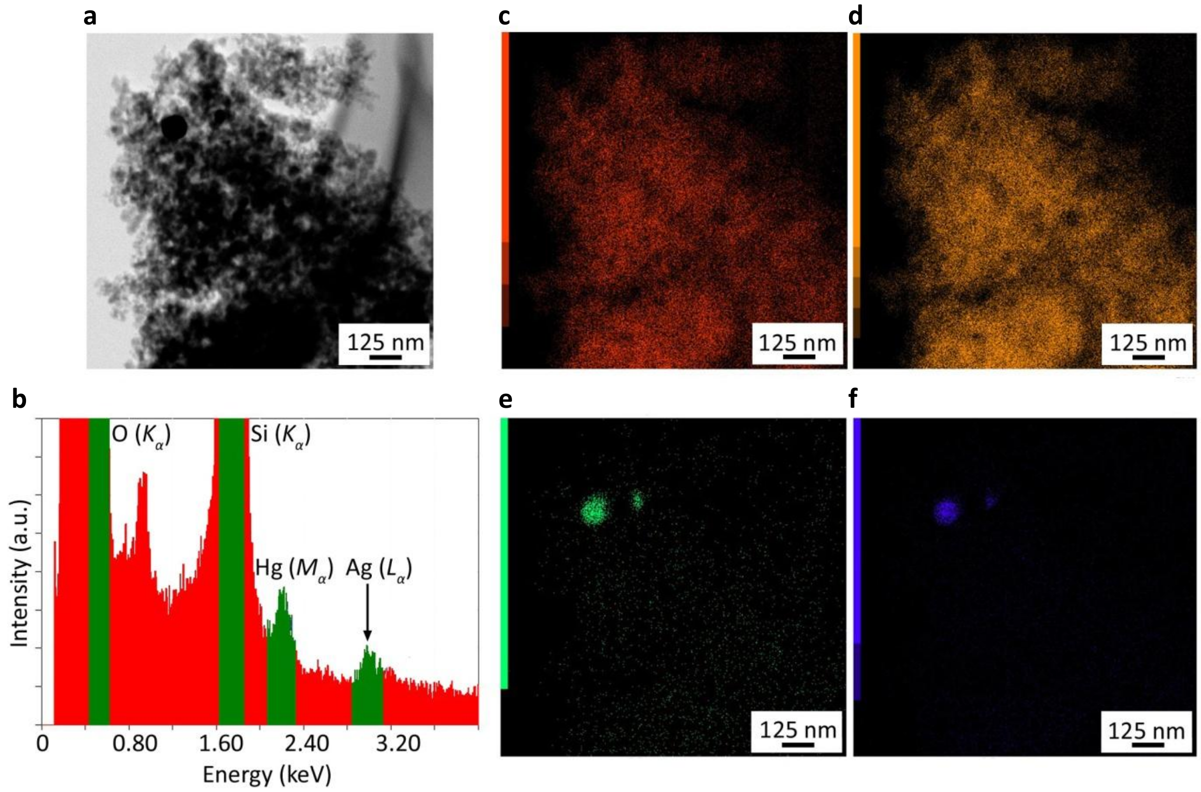


***Figure S1.*** (**a**) TEM image reveals the cluster of silica particles acting as the support site for AgNPs. Due to the contrast of overlapping silica particles it is difficult to discern the location of AgNPs, therefore (**b**) EDX analysis was performed to reveal the chemical composition of the sample. EDX mapping analysis can then be applied to the TEM image to reveal the distribution of elements within the sample to show the location of (**c**) Si ($K_α$ peak), (**d**) O ($K_α$ peak), (**e**) Ag ($L_α$ peak) and (f) Hg ($M_α$ peak). Mercury is only found on the sites corresponding to the location of AgNPs.

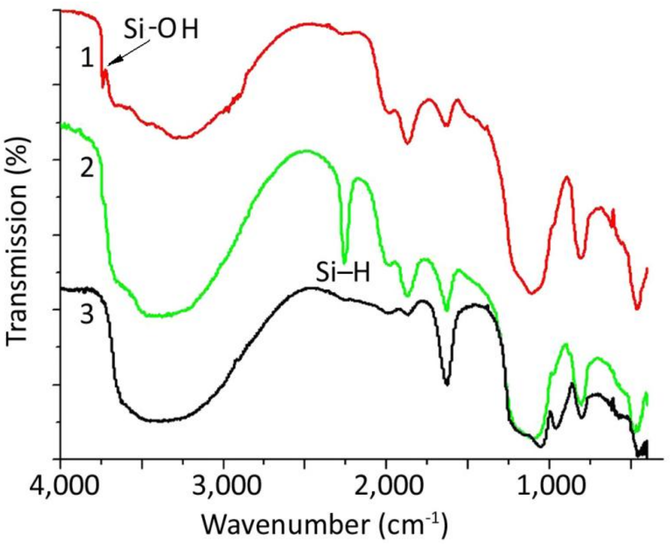


***Figure S2.*** FTIR spectra of (1) silica reveal the generation of (2) silicon hydride groups and (3) their conversion to silanol groups in the presence of silver nitrate solution resulting in the formation of AgNPs.

After incubation with silver nitrate, AgNPs are generated on the surface of silica. Their presence was confirmed by TEM, EDX analysis (Fig. S1) and powder X-Ray Diffraction (Fig. 3a). Analysis of the (111) peak [1] shows AgNPs with a mean diameter of 11 nm, 31 nm and 45 nm for C-120-Ag-5, C-120-Ag-10 and C-120-Ag-44 respectively. Therefore, the concentration of silicon hydride groups and silver nitrate, plus the incubation time, controls the final size of the metal nanoparticle.

## S3. Removal of aqueous mercury

In a control test, at pH 4-7 the unmodified samples of C-120 absorb less than 1.09 x $10^{-4}$ mmol Hg / g $SiO_2$, which shows that mercury is not removed by silanol group or by the reaction vessel. The interaction of Hg only occurs at the location of AgNPs, as shown by EDX mapping analysis (Fig. S1d-f), which results in the formation of an amalgam. This is accompanied by the disappearance of the crystal lattice structure (Fig. 3c to 3e) and an increase in particle size (Fig. S3), which for 11 nm AgNPs increases to an average diameter of 44 nm (Fig. S4). The initially narrow distribution of AgNP size broadens with the formation of the amalgam, though TEM images reveal that a few particles have fused or additional Ostwald ripening processes may have occurred yielding larger than expected particles (Fig. S3) given that for the 1.25 : 1.00 system, the particle was calculated to increase from 11 to 24 nm rather than the observed 44 nm.

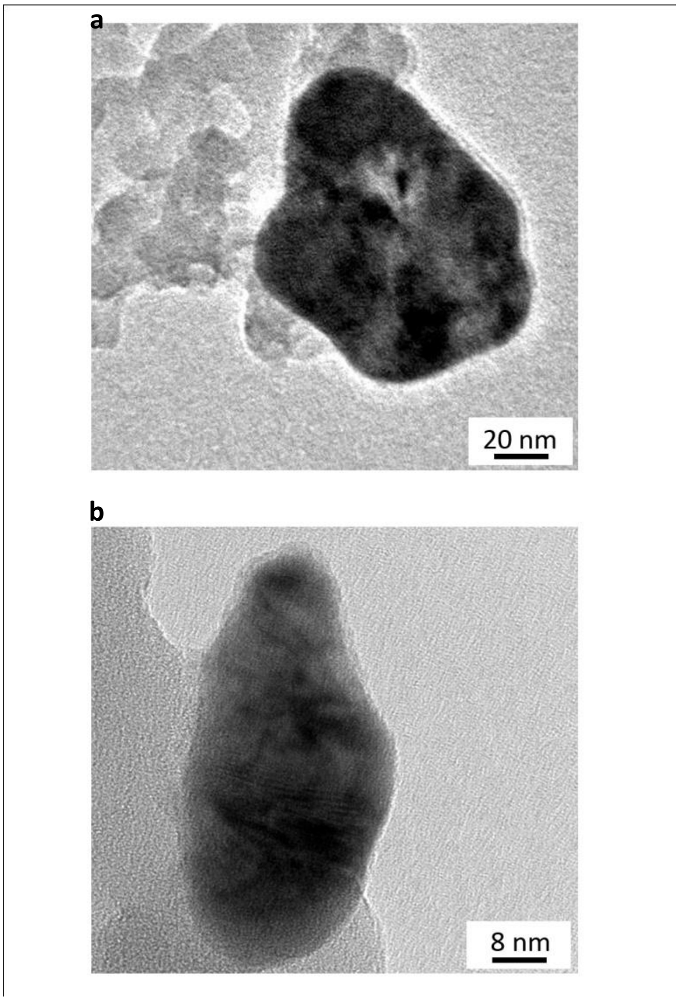


***Figure S3.*** Particle size was determined from TEM images before and after formation of the amalgam, which revealed (a) a number of particles that appear to have aggregated and (b) particles that have grown in size, leading to a larger average particle size for the Ag-Hg amalgam.

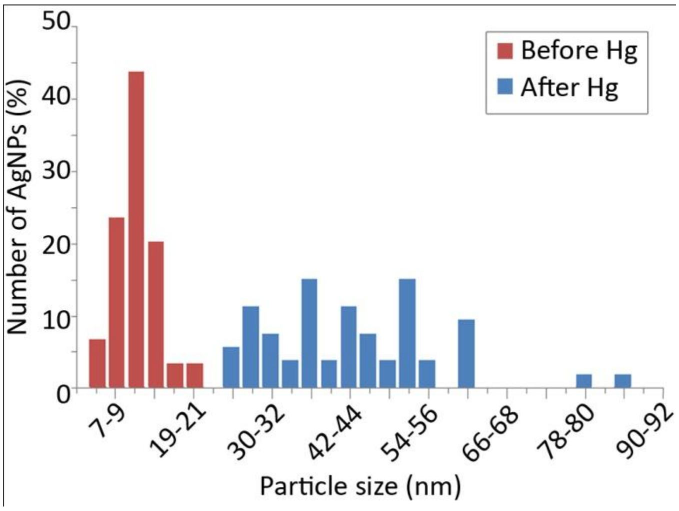


***Figure S4.*** Particle size was determined from TEM images before and after formation of the amalgam, which revealed a larger average particle size for the Ag – Hg amalgam.

XPS analysis was performed on C-120-5 samples (11 nm AgNPs) before and after contact with mercury. The initial AgNP on silica sample reveals two peaks for silver at 367.6 eV and 373.6 eV (Fig. S5a), corresponding to the $3d_{5/2}$ and $3d_{3/2}$ of metallic silver respectively. These peaks are slightly shifted by around -0.5 eV from bulk metallic silver, which may result from the reduced particle size or the interaction of AgNPs with the oxygen part of the silanol groups. After reaction with mercury, the intensity of both peaks is reduced, however, there is no shift in peak position or emergence of new peaks, which suggests its reduced presence in the XPS beam, possibly due to its disaggregation from the (111) crystal phase on contact with mercury. This is supported ICP-MS analysis of the solution after adsorption experiments (which reveals less than $1 * 10^{-3}$ mmol of silver in solution) and the dissolution experiments (revealing at least a 0.7 : 1 ratio of Hg to Ag in the final system), indicating that silver is retained in the solid form. Therein, the oxidation state of silver remains the same throughout the experiment.

The use of silica as a support substrate leads to a characterisation difficulty in the analysis of the mercury oxidation state, where the Si 2p of silica has a broad binding energy peak centred around 103.1 eV (Fig. S5b), which overlaps the possible presence of Hg 4f $_{5/2}$ and 4f $_{7/2}$ peaks expected at 105.8 and 101.7 eV respectively for mercury (II). Curiously the peak at 99.8 - 100.2 eV for metallic Hg is not discernible in the tail region of the Si 2p peak, which implies that mercury (II) has not been reduced to its elemental state. However, the XRD profile clearly reveals that the schachernite (amalgam) crystal phase has formed during the course of the reaction, therefore mercury must have reduced. And given that EDX analysis shows the elemental ratio of Hg to

Ag is around 1 : 1, whereas ICP-MS for the adsorption and the dissolution experiments of the same sample have a ratio of 0.7 : 1, infers that mercury is leaving the system during its preparation for TEM-EDX analysis. Therein, vaporization of mercury from its silver amalgam within a vacuum occurs. This strongly suggests that mercury is behaving in the metallic state and therefore will not be retained under high vacuum XPS analysis either, hence the absence of metallic peaks in Fig. S5b. Ultimately, mercury (II) is reduced by AgNPs without the oxidation of silver ensuing and occurs with hyperstoichiometry ratios.

We compared the adsorption kinetics for all AgNPs on silica samples, but due to the incubation and subsequent separation times required, data points at less than 3 minutes could not be obtained. However, 95 % of mercury (II) was removed from solution within this timeframe, denoting very fast adsorption kinetics (Fig. 2c). AgNPs with 11 nm diameter exhibit the fastest rate of Hg (II) adsorption, which is surmised to occur due to AgNPs with the smallest diameter possessing the largest surface area and shortest diffusion length among the samples tested. The release of Ag (I) into solution from AgNPs in contact with Hg (II) solution promotes reduction of mercury to a zero-valent state Equation (1), and the latter can form an amalgam with the metallic silver in the nanoparticles. However, the molar mercury-to-silver ratio far exceeds that of the bulk scale redox reaction.

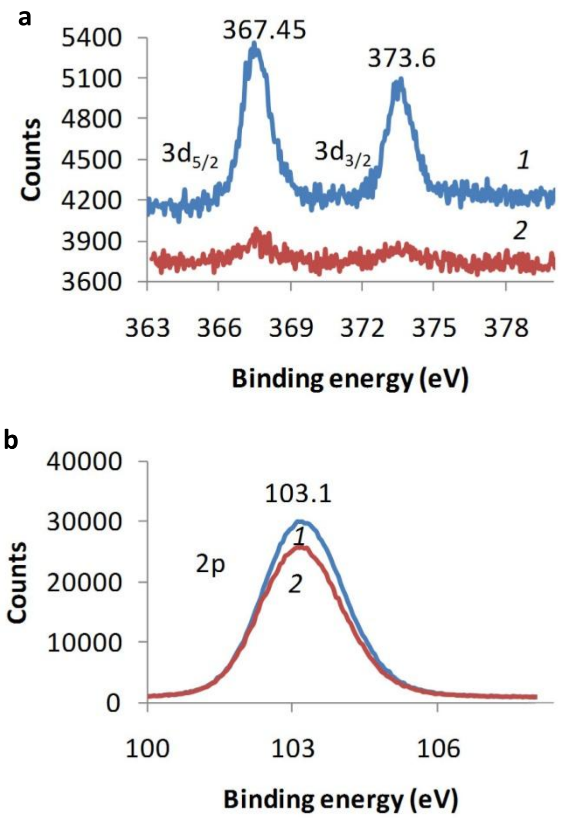


***Figure S5.*** XPS reveals the binding energy of (a) silver in the metallic state and (b) the broad peak of Silica 2p (which overlaps any mercury peaks present) for (1) before and (2) after reaction of AgNPs with $Hg^{2+}$.

Mercury as Hg (0) and Hg (II) are the most common states in nature.[2][3] Despite significant interest in mercury speciation in the natural environment due to its toxicity, its environmental chemistry is not well understood. In general it is assumed that metallic mercury, Hg (0), in water prevails under reducing or mildly oxidising conditions, unless sufficient sulfide is present to stabilise the resulting complex or bivalent mercury. Under strongly reducing conditions, Hg(II) converts to Hg(0).

Hg (II) can also be reduced to Hg (0) photochemically and according to Alberts et al. mercuric ions ($Hg^{2+}$) can be reduced in aqueous solutions by humic acids, which is thought to be due to the interaction with the free radical electrons of the humic acid.[4]

In the present context, the redox reactions (Equation S1) and (Equation S2), in which Hg (II) nitrate converts into Hg (0), are thermodynamically favourable with ΔG of -11.32 and -48.12 kJ/mol respectively:

$Hg^{2+}(aq) + 2\ NO_3^-\ (aq) + H_2O\ (l) = Hg^0\ (l) + 2\ H^+\ (aq) + 2\ NO_2^-\ (aq) + 3/2O_2\ (g)$ (S1)

$\Delta G_{r1} = -\ 11.32$ kJ/mol

$Hg^{2+}(aq) + 2\ NO_3^-\ (aq) + H_2O\ (l) = Hg^0\ (l) + 2\ HNO_2\ (aq) + 3/2O_2\ (g)$ (S2)

$\Delta G_{r2} = -\ 48.12$ kJ/mol

Thermodynamic calculations do not, however, predict the rate of such transformations, and a mercury (II) nitrate solution stored in dark is stable. It is therefore likely that a redox active anion, such as nitrate and acetate, may facilitate the reduction of silver released into solution under conventional redox chemistry (according to equation 1) and allow silver to partake in further reduction of mercury.

## S4. Physicochemical properties of AgNP adsorption

The chemical binding affinity of a sorbent can be expressed in terms of the distribution coefficient ($k_d$). The higher the $k_d$ value, the more effective the sorbent material is at capturing and holding the target species. The distribution coefficient ($k_d$) (in mL/g) is the mass-weighted partition coefficient between the liquid supernatant phase and solid phase according to equation (S3):

$$k_d = \frac{(C_0 - C_f)}{C_f} \frac{V}{m} \qquad \text{(S3)}$$

where $C_0$ and $C_f$ are the initial and final concentrations in the initial and final concentrations in the solution of the target species determined by ICP-MS, $V$ is the solution volume in millilitres, and $m$ is the mass in grams of the sorbent. The value of $k_d$ for the silica with chemically immobilised β-cyclodextrin is not higher than 100 ml per g.[5] The mercury loading distribution coefficient ($k_d$) is 5500 ml per g for C-120-Ag-10 at pH 7.

The formation of AgNPs generates an absorption band around 400 nm caused by the collective oscillation of conducting electrons on the surface of the metal, thus giving a yellow appearance. The UV-Vis spectral response also changes with increasing concentration of mercury adsorbed on to AgNPs, where the absorption band corresponding to the surface plasmon energy of Ag around 390 nm is reduced in intensity and is accompanied by a hypsochromic shift toward around 365 nm (Fig. S6a). When the concentration reaches 39 mg per litre the surface plasmon band in the UV-Vis spectrum almost disappears. Notably, the colour of the samples changes with increasing quantity of adsorbed mercury (Fig S6b). The linear dependence of change of optical density of silver-mercury amalgam with respect to the concentration of mercury adsorbed (Fig. S6a insert) may be useful in the rapid and facile detection of high levels of Hg (II) in monitoring mining, industrial effluent and chemical processing environments, though we recognise that if the mechanism is dependent on a redox active anion species, its application becomes limited.

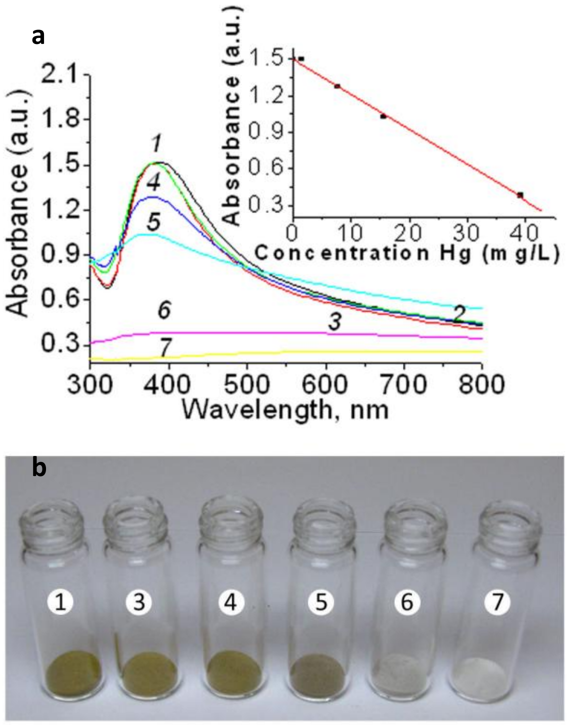


***Figure S6.*** UV-Visible diffusion spectra of C-120-Ag-10 samples before (line 1) and after contact with $Hg(NO_3)_2$ solution of increasing concentration 0.15 (line 2), 1.56 (line 3), 7.81 (line 4) 15.6 (line 5) 39 (line 6) to 78 (line 7) mg per litre. The reducing intensity of the 390 nm peak accompanies the increasing adsorption of mercury, which is used to derive a calibration curve (insert).

**Conclusions**

The feature of hyperstoichiometry has significant potential in numerous applications. In one embodiment, the system demonstrates a marked improvement in the adsorption of Hg (II) over that of bulk-scale silver indicating the potential application of size-controlled AgNP-based composite devices in the removal of Hg from wastewater and industrial effluents, where the appropriate anions are present. The immobilisation of AgNPs to silica substrates lends itself to the separation of AgNPs, which in turn will be useful for rapid decontamination in a through-flow device configuration. This route provides a safer and quicker mercury removal than the commonly employed precipitation and adsorption techniques. As an example using a previous case study for mercury decontamination,[6] to treat 113.6 ML of 15 ppb Hg(II) under conventional redox mechanism would require 21,888 g of silver rods (10 micron diameter); in contrast, for an equivalent treatment, only 3,251 g of 45 nm Ag and only 811 g of 11 nm Ag (on silica) are required owing to the previously unknown

hyperstoichiometry effect reported in this paper. Therefore, AgNP/silica composites can be placed in a significantly smaller volume treatment unit at a lower bulk adsorbent material cost.

## References


[1] J. Barkauskas, S. Budriene, A. Dervinyte, *Journal of Analytical and Applied Pyrolysis* **2004**, *71*, 709.

[2] Lindsay, W. L. Chemical Equilibria in Soils. New York: John Wiley& Sons, pp. 343-363 (1971).

[3] Andersson, A. Mercury in Soils. In: Nriagu, J.O. (cd.), The Biogeochemistry of Mercury in the Environment. Amsterdam: Elsevier. pp. 79-112 (1979).

[4] Alberts, J.J., Schindler, J.E., Miller, R.W. & Nutter, D.E. Elemental mercury evolution mediated by humic acid. Science 194, 895-896 (1974).

[5] L. A. Belyakova, D. Y. Lyashenko, A. N. Shvets, Zhurnal Fizicheskoi Khimii 84, 741 (2010)

[6] Treatment Technologies for Mercury in Soil, Waste, and Water, U.S. Environmental Protection Agency, Washington (2007).
